# Comparative evaluation of occlusal forces in altered and unaltered VDO using OccluSense

**DOI:** 10.6026/973206300214912

**Published:** 2025-12-15

**Authors:** Metorilang Sohlang, Pavan Kumar Dubey, Rajesh Bansal, Mahesh Khairnar, Vinay Srivastava

**Affiliations:** 1 Department of Prosthodontics, Crown & Bridge and Oral Implantology, Institute of Medical Sciences, Banaras Hindu University, Varanasi, Uttar Pradesh, India; 2 Department of Public Health Dentistry, Institute of Medical Sciences, Banaras Hindu University, Varanasi, Uttar Pradesh, India; 3 Department of Pediatric and Preventive Dentistry, Faculty of Dental Sciences, Institute of Medical Sciences, Banaras Hindu University, Varanasi, Uttar Pradesh, India

**Keywords:** Occlusal forces, vertical dimension, full mouth rehabilitation

## Abstract

Tooth wear alters occlusal forces and vertical dimension, making accurate assessment essential for effective full-mouth rehabilitation. Therefore, it is of interest to evaluate occlusal force distribution in 65 subjects aged 35-60 years using OccluSense, including controls and patients with attrition having altered or unaltered VDO. Statistical analysis with ANOVA and t-tests showed no overall significant intergroup differences, though Group 2 (attrition with altered VDO) demonstrated significantly higher forces in the right anterior region. Intragroup analysis also revealed significant forces in the right anterior and right first molar regions. Thus, digital occlusal analysis is valuable for precise diagnosis and treatment planning in full-mouth rehabilitation patients.

## Background:

The stomatognathic system functions as an integrated biomechanical unit in which harmony among teeth, periodontal structures, temporomandibular joints, and neuromuscular components is essential for efficient mastication and oral function [1]. Occlusal harmony is achieved when there are no interceptive or deflective contacts in maximal intercuspation or during eccentric mandibular movements, allowing uniform force distribution without overloading the system [2]. Occlusal force magnitude is influenced by tooth morphology, number and configuration of roots, periodontal ligament health, alveolar bone support, and total occlusal surface area [3]. Bite force is further determined by jaw elevator muscle efficiency, mandibular lever mechanics, occlusal contact pattern, and anthropometric factors such as height, weight, and craniofacial dimensions [4]. The molar region exhibits the highest occlusal force because of its favorable mechanical advantage and proximity to the temporomandibular joint [5]. In individuals with sound dentition, unilateral maximum bite force in the molar region typically ranges between 300 and 600 Newtons [6]. With increasing age, occlusal anatomy may be altered by physiological wear, mechanical attrition, chemical erosion, parafunctional habits such as bruxism and clenching, gastroesophageal reflux disease, and developmental abnormalities [7]. Turner and Missirlian, in 1984, classified severe tooth wear into three categories based on the presence or absence of VDO loss and the availability of restorative space, providing a clinically relevant framework for diagnosis and treatment planning [8]. Patients within these categories frequently require comprehensive full mouth rehabilitation to restore occlusal function, esthetics, and biomechanical harmony [9]. Digital occlusal analysis systems like Occlusense allow objective quantification of occlusal force distribution; however, evidence using this technology in patients classified according to Turner and Missirlian remains limited [10]. Therefore it is of interest to study the use of digital occlusal analysis for the evaluation of the forces during occlusion.

## Materials and Methods:

## Patient selection:

65 subjects in total were chosen according to the inclusion and exclusion criteria and allotted into 3 groups. 20 volunteers (12 males, 8 females) were included in Group 1 (Control- completely dentate subjects without any attrition). 45 study subjects were allotted according to Turner and Missirlian Classification 1984 into two groups: 20 patients (15 males, 5 females) in Group 2 (T & M category 1) and 25 patients (6 females, 19 males) in Group 3 (T & M category 2, 3).

## Methodology:

After careful explanation, written informed consent was obtained. Questionnaires were formulated and clinical examination of the subjects was performed. For study groups, adhesive micropore tape (3M Micropore, India) was applied on the nose tip and chin prominence. A cross sign was marked on the tape as a fixed reference point for measurement of both the vertical dimension at rest (VDR) and at occlusion (VDO). VDR was measured using phonetic method and physiologic method where VDO was obtained by guiding the patient to close his teeth in maximal intercuspation. The Interocclusal distance (IOD) was then derived from the difference between VDR and VDO. The study subjects were then allotted into Group 2 and 3 as stated above.

## Recording of occlusal forces:

The subjects sat upright with their head unsupported, ensuring the Frankfort plane was nearly parallel to the floor. They were trained on sensor placement and force measurement before recording. Bite force was captured using Occlusense sensors (Bausch, Germany) after establishing a Wi-Fi connection with the system. Patient details were entered into the Occlusense app on an iPad (Apple Inc), activating the system. After sensor placement, patients were asked to bite in maximal intercuspation. Recording started by pressing a pink button, capturing static bite force at 100 Hz for 4 seconds. This was repeated three times to ensure accuracy. The sensor was then discarded, and the handset disinfected. The highest force recorded between 2-3 seconds was used for the reading.

## Statistical analysis:

SPSS v23.0 software was used for data analysis. 5% was kept as the level of significance. One-way ANOVA test was used for intergroup occlusal forces comparison. Intragroup forces for the study groups were compared using an independent t-test.

## Results:

Recordings for all groups were tabulated. Statistical analysis is represented in Graphs 1-3. Group 2 exerted more force than Groups 1 and 3. However, the right side's total force was statistically significant only in the anterior region (p=0.041) (Figure 1 - see PDF). No statistical differences were found for left side or combined total force. Intragroup analysis showed Group 2's total force between right and left sides was significant in the anterior (p=0.037) and first molar (p=0.047) regions. Combined total force was also significant (p<0.001) (Figure 2 - see PDF). However, no statistical difference was found in Group 3's intragroup analysis. Figure 3 (see PDF) revealed that in Group 1, the cumulative force from posterior teeth was significantly greater than from anterior teeth on both sides. In Group 2, the right side exhibited greater force from the anterior teeth, while the left side revealed the opposite. In Group 3, anterior teeth exerted lower force than posterior teeth, but differences were insignificant.

## Discussion:

Occlusal disease, linked to ageing causes interferences, premature contacts leading to disharmony. This study found occlusal diseases were common in the 35-60 age groups. Van't Spijker reported that tooth-wear increased from 3% at age 20 to 17% at age 70 [4]. Mameno *et al.* found a significant association between dietary hardness, occlusal force and contact area, suggesting younger participants with intact tooth anatomy chewed better than older ones [5]. EMG measurements showed greater masticatory muscle activity in younger subjects, leading to higher occlusal forces. A non-veg diet, being coarser, requires more chewing, increasing tooth-wear. The low pH of acidic meals and beverages also causes erosion of enamel and dentin. Tooth-wear is influenced by bristle type, brushing method, duration, frequency, and cleansing agent. Many subjects used "Gul Manjan" and "Lal Dant Manjan," tobacco-based dentifrices common in rural India and Bangladesh which are carcinogenic and addictive. Their use, along with twig brushing, led to severe tooth-wear, decreased vertical dimension, dentin and pulp exposure, gingival recession, and poor oral hygiene. These findings were supported by Shah *et al.* [6]. Gastroesophageal reflux disorder (GERD) and eating disorders like bulimia contribute to tooth-wear. GERD presents a characteristic erosion of the lingual surface of molars and bulimia causes enamel erosion on the palatal surfaces of the upper anteriors [7]. Anxiety and depression often coexist and significantly impact overall health, with fatigue and psychological distress being common in today's working population. However, higher literacy rates have been linked to a reduction in these conditions. The present study aligns with AM da Silva *et al.*'s findings, showing that patients with tooth-wear exhibited more anxiety traits, suggesting faster tooth-wear could be an indicator of persistent anxiety [8]. Parafunctional habits like bruxism and object biting contribute to tooth-wear. The American Academy of Sleep Medicine identifies tooth-wear as a diagnostic feature of sleep bruxism, often linked to sleep apnoea [9]. Christensen GJ found that bruxists grind their teeth in eccentric positions [10]. Diraçoglu *et al.* observed higher tooth-wear and increased incisor MBF in bruxists [11]. Cosme *et al.* noted similar MBF at the molar region in both groups [12]. Pizolato *et al.* found reduced MBF in bruxist women, attributed to a lower pain threshold in women [13]. However, Baba *et al.* failed to show an association between bruxism and tooth-wear [14]. Jaw clenching also contributes to tooth-wear; clenchers often bite into centric occlusion. Christensen GJ observed that clenchers had severe anterior wear than posterior wear with steep incisal and canine guidance [10]. Clenching could be an objective marker to diagnose psychological distress. Bracha *et al.* observed that it could enhance blood circulation to the temporal lobe, especially in acutely distressed individuals, and that incisor wear is common in patients with Post-traumatic Stress Disorder (PTSD) [15]. Temporomandibular disorders (TMDs) are linked to increased tooth-wear, particularly in females. Oginni *et al.* reported that patients with TMDs demonstrated higher tooth-wear in posterior teeth along with TMJ sounds and pain [16]. Several studies report decreased bite force in TMD patients due to articular pain, limiting elevator muscle contraction as a protective mechanism. The study also reported joint pain, likely related to the subjects' occupations as farmers. TMDs are associated with cervical pain with stiff neck being a symptom of occlusal disease. Khan *et al.* associated occlusal disease signs to posture and TMDs. A neuromuscular connection links the TMJ to cervical and scapular regions, with TMD patients often showing upper cervical hyperextension and lower cervical spine straightening [17]. The present study utilized VDO for subject categorization. For this, Turner and Missirlian classification 1984, most popularly used for rehabilitation of worn-out dentition was followed [1]. Most study subjects belong to T & M category 2, 3. This may be because vertical dimension loss of the worn-out dentition is always compensated by dentoalveolar elongation to maintain the consistent repetitive contractile length of the elevator muscles [7]. Few studies have used the Occlusense system for force measurement. The handle captures data at 0.056-second intervals using a 60µm sensor with red articulating ink. Based on "Force Sensing Resistor" mechanism, applied force compresses the resistor between silver electrodes, reducing resistance. After optimization, recordings were set at 100 Hz for 4 seconds during static maximal intercuspation. Data is displayed in 2D and 3D with color-coded pressure gradients- green (minimum) to red (maximum). Red indicates interferences or initial contact; green indicates uniformly abraded or planar contacts. The system also shows twelve zones and quartered force percentages [18]. The study observed that males have increased tooth-wear than females which was consistent with various studies. Half of the patients included in Group 2 had deep bite with palatally upper anterior and labially lower anterior tooth-wear. This results in steep anterior guidance that interferes with the envelope of function [7]. According to Cunha *et al.* children and adolescents with class 2 malocclusion and no posterior or anterior open bite had increased tooth-wear than adults [19]. The study observed a prevalence of anterior over posterior tooth-wear and increased wear in group function than canine-guided occlusion. This is supported by Johansson *et al.* [20]. This may be because incisors have thinner enamel; they along with canines are involved in masticatory and excursive movements, both in function and parafunction [7]. In ageing and occlusal diseases, canine guidance is replaced by group function. Considering the study's aim, no significant difference in occlusal forces was observed among the three groups, contrasting with Jain *et al.* [21] who found significantly lesser force in subjects with moderate to severe attrition-though their measurements were limited to the first molar region only. Kiliaridis *et al.* reported higher bite force in subjects with occlusal wear (600N) compared to controls (400-450N) [22] possibly due to parafunction or greater tolerance in muscle contraction mechanisms enhancing masticatory muscle function. Subjects with altered VDO exerted greater forces on the right side anterior region than the other groups. Additionally, intragroup analysis of the said group revealed significant forces at both right anterior and right first molar regions, potentially related to the subjects' dominant chewing side. Khan *et al.* confirmed that the dominant chewing side showed higher mean maximal voluntary bite force than the non-dominant side in both genders [23]. Additionally, posterior bite force was greater than the anterior in both sides of the control group, consistent with Gözen & Güntekin [1] who noted that in normal dentition, the posterior region exhibits the strongest association between occlusal contact and the maximum bite force. The molars exert a higher occlusal force mainly because of the mandibular lever system and the jaw elevator muscles' biomechanics. The questionnaires also revealed that while cosmetic considerations hold significance, patients tend to prioritize optimal oral function above all else. The limitations of the study includes- small sample size, also qualitative, quantitative tooth-wear, force comparison among genders, occlusal scheme, malocclusion and dominant chewing side were not considered. Further research may be considered taking into account the dominant chewing side and bite force pattern of patients with worn-out dentition.

## Conclusion:

Digital measurement of occlusal forces provides valuable insights for full-mouth rehabilitation, supporting accurate diagnosis and well-planned prosthodontic interventions. Although overall force differences between study and control groups were not significant, altered VDO cases showed notable regional variations, particularly in the right anterior and right first molar areas. Thus, the need for precise occlusal force evaluation to enhance treatment outcomes in patients with VDO discrepancies is shown.

## Funding:

Institute of Eminence, BHU

## Disclosure forms:

## Source of support or funding:

Institute of Eminence, BHU

(Grant No.- R/Dev/D/IoE/Seed Grant/2020-21/ Dated-July 1 2020 Under Development Scheme No. 6031)

## CRedIT authorship contribution details:

Metorilang Sohlang: Definition of intellectual content and visualization, investigation/data collection, manuscript writing, resources apart from funding

Pavan Kumar Dubey: Conceptualization, design/methodology, supervision, editing and validation

Rajesh Bansal: Supervision and validation

Mahesh Khairnar: Statistical analysis and validation

Vinay Srivastava: Supervision and validation

## Ethical policy and Institutional Review board statement

(submitted under supplementary files)

Institutional Ethics Committee, Banaras Hindu University, Varanasi ECR/526/Inst/UP/2014/RR-20 dt.19.5.2020 No.Dean/2022/EC/3462

Registration under Clinical Trial Registry (submitted under supplementary files): CTRI/2024/03/063768.
